# Immersive virtual reality for shoulder rehabilitation: evaluation of a physical therapy program executed with oculus quest 2

**DOI:** 10.1186/s12891-023-06861-5

**Published:** 2023-11-02

**Authors:** Umile Giuseppe Longo, Arianna Carnevale, Flavia Andreoli, Ilaria Mannocchi, Marco Bravi, Mohamed Saifeddine Hadj Sassi, Fabio Santacaterina, Marco Carli, Emiliano Schena, Rocco Papalia

**Affiliations:** 1 Fondazione Policlinico Universitario Campus Bio-Medico, Via Álvaro del Portillo, Roma, 200, 00128 Italy; 2 Research Unit of Orthopaedic and Trauma Surgery, Department of Medicine and Surgery, Universit? Campus Bio-Medico di Roma, Via Álvaro del Portillo, Roma, 21, 00128 Italy; 3 Department of Industrial, Electronic and Mechanical Engineering, University of Roma Tre, Via Vito Volterra, Roma, 62, 00146 Italy; 4 Research Unit of Physical and Rehabilitation Medicine, Fondazione Policlinico Universitario Campus Bio-Medico, Via Álvaro del Portillo, Roma, 200, 00128 Italy; 5 Department of Movement, Human and Health Sciences, University of Rome “Foro Italico”, Roma, 00135 Italy; 6 Laboratory of Measurement and Biomedical Instrumentation, Department of Engineering, Università Campus Bio-Medico di Roma, Via Álvaro del Portillo, Roma, 21, 00128 Italy

**Keywords:** Shoulder, Rehabilitation, Virtual reality, Immersive virtual reality, Usability, Acceptability, IV

## Abstract

**Background:**

Virtual Reality (VR) systems have been increasingly used across several medical fields. A crucial preliminary step for developing optimized VR-based applications for rehabilitation purposes is identifying potential interventions to meet the requirements necessary to satisfy end-users’ needs. This study aims to assess the acceptability, usability, and appropriateness of a VR physical therapy program executed with Oculus Quest 2 by expert physiotherapists of shoulder musculoskeletal rehabilitation.

**Methods:**

Eleven physiotherapists were enrolled to test a VR program for shoulder musculoskeletal rehabilitation. At the end of each session, physiotherapists completed three questionnaires about the acceptability, usability, and appropriateness of the VR system and application, investigating aspects such as wearability, safety, stability, ease of control, comfort, size, utility, playability, and use mode.

**Results:**

The acceptability questionnaire revealed that all the physiotherapists found the VR system easy to wear and control, very confident, and safe. The usability questionnaire showed that most physiotherapists (73%) found the VR application entertaining, although only 45% said the system could be used independently by patients without the support of a therapist. Many physiotherapists found the use of the VR application appropriate for patients with rotator cuff tears treated conservatively (63.6%) or surgically (54.5%), for patients with shoulder osteoarthritis treated conservatively (72.7%), for patients with shoulder osteoarthritis after surgical treatment (63.6%). 91% of physiotherapists think it would be best for patients to use the VR system under the supervision of a therapist and not independently in a home setting.

**Conclusions:**

The use of VR in orthopaedic rehabilitation is encouraging, although further efforts are needed to increase the independent use of patients without the supervision of a physiotherapist. Moreover, future studies should strive to ensure the clinical effectiveness of VR rehabilitation in reaching therapeutic goal settings.

## Introduction

Cutting-edge technological solutions (e.g., virtual reality (VR), augmented reality, telerehabiitation) have recently been introduced into the orthopedic rehabilitation field to make it more effective and personalized [1–3]. VR is a computer-generated 3D environment that makes highly interactive experiences possible, significantly impacting patient entertainment, education, and communications [1]. VR has been increasingly used in various medical fields, including mental health therapy [4–6], neurological rehabilitation [7–9], low back pain [10, 11], and shoulder disorders [12–14]. Moreover, VR has been applied for educational purposes simulating surgical procedures in both orthopedic and neurological fields [15–17].

Recent studies showed that VR-based systems could be an optimal support element for increasing patients’ motivation and engagement in the prescribed rehabilitation programs [13, 14, 18, 19]. Few contributions are currently available in the literature on shoulder rehabilitation supported by VR systems in patients suffering from rotator cuff tears, frozen shoulder, or subacromial impingement [14, 18, 20]. However, there are some studies in the literature that have developed VR applications for shoulder rehabilitation in patients with non-orthopaedic conditions, such as patients following breast cancer surgery [21, 22].

Rehabilitation is an intensive process requiring high compliance with therapeutic programs, often affected by limited accessibility, high costs, and lack of engagement [2, 23–25]. In particular, monitoring and guiding patients with shoulder diseases could be of great relevance given the high disability and related rising costs [26–28].

VR systems have the potential to provide engaging scenarios, and realistic and multi-sensory experiences, resulting in a stimulating rehabilitation platform for the users, although many aspects (such as safety, use mode, ease of control, wearability, and utility) need further analysis to confirm their usability and effectiveness in shoulder musculoskeletal rehabilitation [29–31].

In an era of rapid technological growth and advances in digital technology applied to medicine, many efforts are needed to establish the real effectiveness and practical utility of applying emerging VR solutions [32, 33]. Among VR systems, very few investigations have dealt with the application of VR systems in the context of shoulder musculoskeletal disorders [12, 13]. Therefore, further research investigating the required features of up-to-date VR technology for supporting shoulder physical therapy is essential.

Among the most emerging VR systems is Oculus Quest 2 (OQ2) [12, 34]. Recently, the accuracy of the OQ2 in measuring translational and rotational displacements has been evaluated, showing that OQ2 is a promising VR tool for monitoring shoulder kinematics during rehabilitation [34]. The inside-out movement tracking makes OQ2 unobtrusive and wireless, a viable support for traditional management of musculoskeletal shoulder rehabilitation.

A necessary step for future VR-based application improvements is the identification of potential aspects to be changed and enhanced. VR applications design optimization interventions should be geared toward meeting the requirements necessary to satisfy end-users needs. The value added by this study refers to the presentation of a methodological approach to improve step-by-step the developments of a VR application for shoulder musculoskeletal rehabilitation by considering essential suggestions first from experts in the field before clinical trials can be conducted on patients. For this reason, the expectations and opinions of experienced physical therapists are crucial to highlight what aspects can be modified to increase the usability, acceptability, and appropriateness of devices as adjuvants during traditional rehabilitation [35]. Looking at acceptability, usability, and appropriateness, particularly sub-aspects such as wearability, safety, stability, comfort, size, utility, and use mode, is interesting for developers and end-users as these investigations provide new insights to optimize the design and meet the requirements necessary for a specific application.

This study aims to assess the expectations and opinions of a cohort of physiotherapists about the use of OQ2 as VR system during physical therapy of patients with shoulder musculoskeletal disorders. In particular, this study aims to assess the usability, acceptability, and appropriateness of a custom VR application executed with OQ2.

## Materials and methods

### Design of the study, study setting, and ethical approval

A qualitative study was implemented to explore the overall satisfaction and feasibility of using OQ2 for shoulder rehabilitation. The study was conducted at Fondazione Policlinico Universitario Campus Bio-Medico. Ethical approval has been gained from the local Ethical Committee (protocol code 120/21 OSS ComEt UCBM). All participants were informed about the aim of the study and its protocol before providing the dated and signed informed consent.

### Participants

Eleven physiotherapists (male/female: 5/6, mean 37.9 years old) were enrolled in this study. Participants had to match the following criteria: (1) work experience of at least two years in the rehabilitation of shoulder pathologies; (2) willingness to respond to online questionnaires; (3) signature of the informed consent form. Exclusion criteria for the use of OQ2 were: (1) being tired; (2) needing sleep; (3) having digestive problems; (4) being under emotional stress or anxiety; (5) suffering from flu, headaches, migraines, or earaches; (6) being pregnant. The game experience of physiotherapists was evaluated by asking them about the frequency of use (often, sometimes, never) of video games in general, of VR devices, and specifically of OQ2. All participants were free from visual impairments that could have hindered the VR experience.

### Virtual reality rehabilitation program

In this study, OQ2 (Meta Platforms Technologies) was used as VR system. OQ2 has a Head-Mounted Display (HMD) and two controllers to experience an interactive adventure within a virtual world [34]. A custom application was developed in Unity (Unity, v.2020.17). A virtual rehabilitation program was implemented starting from a reference protocol by the American Society of Shoulder and Elbow Therapists [36].

The VR application was set in a naturalistic scenario where users can interact with virtual objects using the controllers. As shoulder musculoskeletal rehabilitation includes cane-assisted exercises, all tasks were designed to be executed using both hands while holding a stick handle grip stand with controllers positioned at each end.

**Fig. 1:**
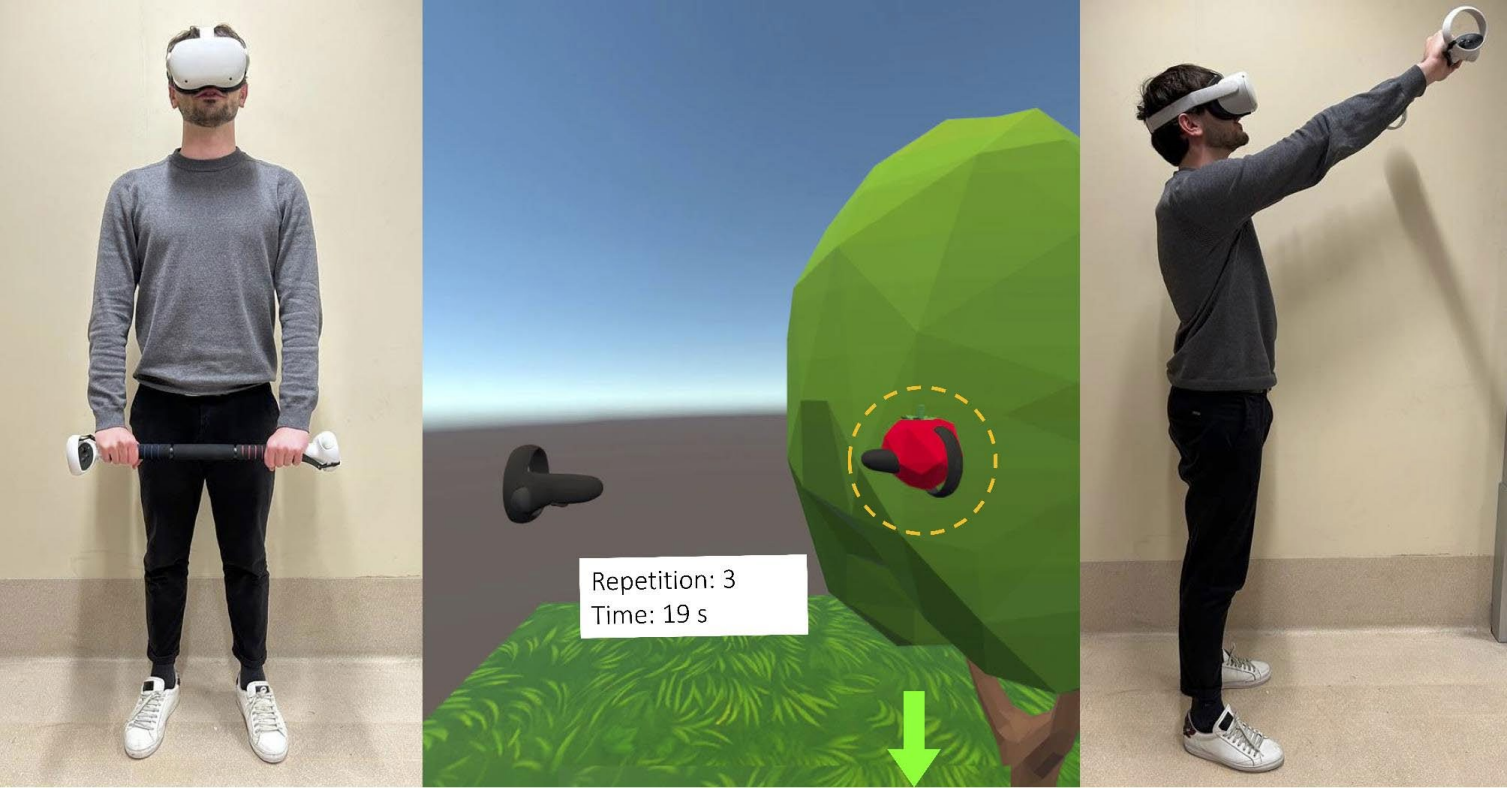
Starting position (left) and final position (right) of the elevation exercise in the sagittal plane. In the center is a representation of what the user visualizes through the head-mounted display and the end effector circled in dotted yellow

The VR rehabilitation protocol is organized into four levels with increasing difficulty in terms of the range of motion (ROM) required to reach a target by simulating the timeline followed during the recovery process in common clinical practice [36]. The first level includes two series of elevations in the sagittal plane in the range 60°-90°, one series of elevations in the frontal plane up to 45°, and one series of extra-rotations up to 20°. The second level includes two series of elevations in the sagittal plane in the range 90°-120°, one series of elevations in the frontal plane in the range 45°-80°, one series of extra-rotations in the range 20°-30°, and one series of intra-rotations up to 20°. The third level includes two series of elevations in the sagittal plane in the range 130°-155°, one series of elevations in the frontal plane in the range 80°-120°, one series of extra-rotations in the range 30°-45°, and one series of intra-rotations in the range 20°-50°. The fourth level includes two series of elevations in the sagittal plane in the range 140°- ROM_MAX_, one series of elevations in the frontal plane in the range 120°- ROM_MAX_, one series of extra-rotations in the range 45°- ROM_MAX_, and one series of intra-rotations in the range 50°- ROM_MAX_. In each level, the exercises are performed consecutively at the end of the previous one. During the elevation in the sagittal plane, players must get an apple from a tree and put it inside a basket (Fig. 1). In this exercise, a movement from top to bottom was required. Apple height increased through levels to simulate achieving a higher goal as synonymous with better ROM skill. Similarly, the second series of elevation in the sagittal plane required performing the movement from bottom to top and with increasing difficulty through the levels.

**Fig. 2:**
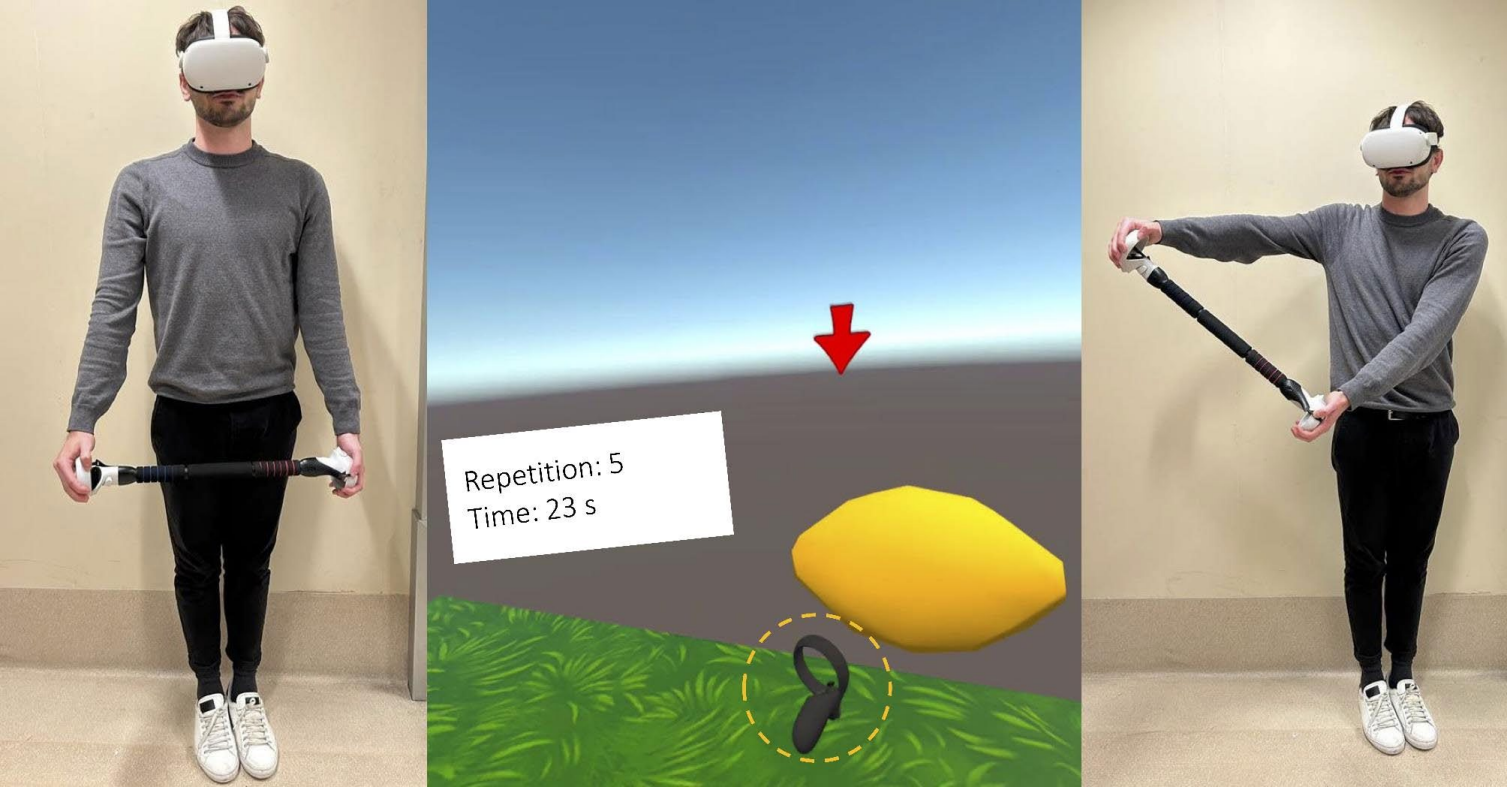
Starting position (left) and final position (right) of the elevation exercise in the frontal plane. In the center is a representation of what the user visualizes through the head-mounted display and the end effector circled in dotted yellow

**Fig. 3:**
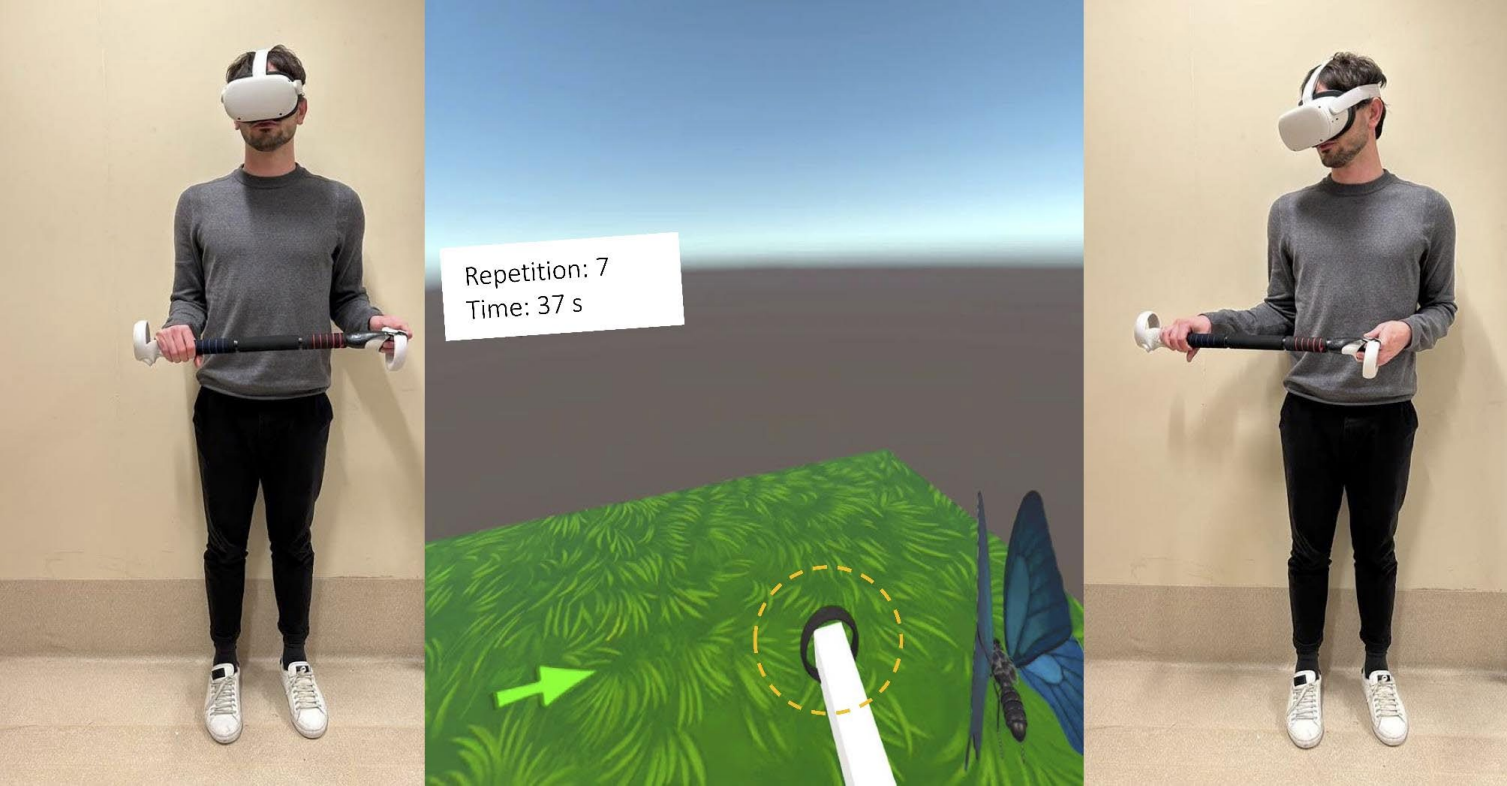
Starting position (left) and final position (right) of the external rotation exercise. In the center is a representation of what the user visualizes through the head-mounted display and the end effector circled in dotted yellow

During the elevation in the frontal plane, players started with the upper limbs along the body and then moved laterally until touching a fruit or vegetable that disappeared after being hit. In this exercise, players performed elevations in the frontal plane until reaching targets gradually placed at increasing heights through the levels (Fig. 2). In the external rotation exercises, players were required to start with elbows flexed to 90° and shoulders adducted. In this position, players were required to grasp a rod to be rotated externally successively until hitting a butterfly (Fig. 3). In the internal rotation exercises, starting from similar conditions, the internal rotation movements were performed in the opposite direction.

### Protocol

The physiotherapists tested the VR rehabilitation program one time. In each session, which lasted about 45 min, physiotherapists executed levels 1 and 4, the easier and the most difficult ones. The study protocol for each participant included the following steps: an explanation for the use of OQ2; OQ2 system calibration; start of the game; training and familiarization in the use of the game; subject height setting; level 1 with elevations in the sagittal plane, elevations in the frontal plane, external rotation exercises; level 4 with elevations in the sagittal plane, elevations in the frontal plane, external and internal rotation exercises.

### Questionnaires

At the end of the experiment, physiotherapists completed online questionnaires with closed and open-ended questions. Two User Experience questionnaires evaluating VR system acceptability and usability were completed [37, 38]. The acceptability questionnaire included 13 questions concerning aspects of device acceptability, namely wearability, safety, stability, ease of control, comfort, size, and general acceptability of the device. Physiotherapists rated statements on a 7-point Likert scale (7 = strongly agree, 4 = neutral, 1 = strongly disagree) [37]. The usability questionnaire included 15 questions classified into three categories, i.e., utility, playability, and use mode [38]. Participants rated statements on a 5-point Likert scale (5 = strongly agree, 3 = neutral, 1 = strongly disagree).

Moreover, an appropriateness questionnaire was developed by the research group, including one open-ended and three multiple-choice questions concerning physiotherapists’ ideas and opinions about the appropriateness of OQ2 and the VR rehabilitation program in the context of patients with shoulder musculoskeletal disorders. In particular, the rationale behind the questions was to understand three additional aspects related to the use of the VR system: for which type of shoulder musculoskeletal pathology and in what period of the rehabilitation is more indicated, and if the supervision of a physiotherapist is required.

## Results

The enrolled physiotherapists had a mean professional experience of 15 years (min-max: 3–25 years, median: 16 years). Most of the physiotherapists (n = 10) were familiar with the use of Video Games, some with the use of VR (n = 3), and just 1 with the use of OQ2.

### Acceptability questionnaire

**Fig. 4:**
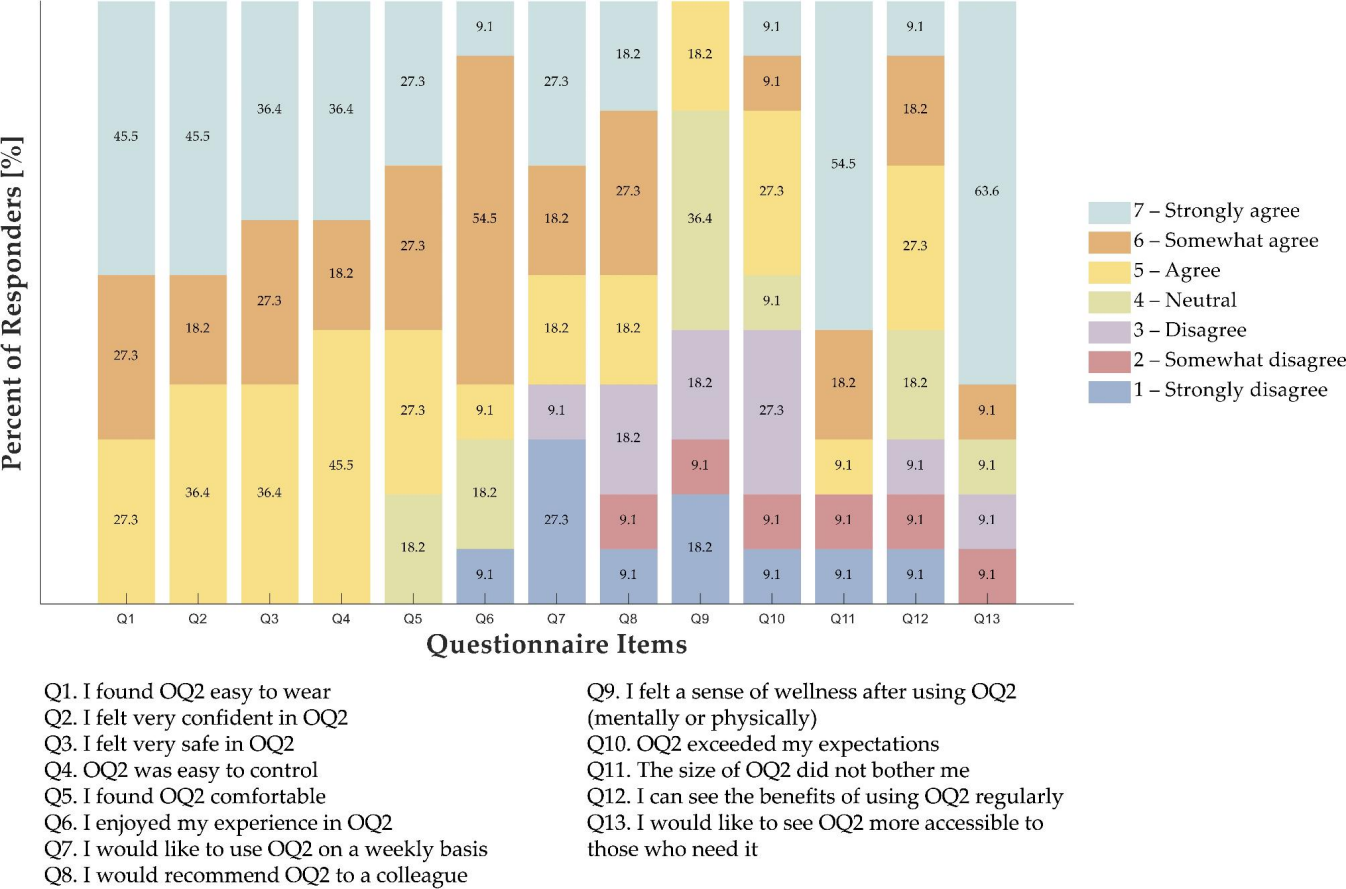
Physiotherapists’ ratings on the acceptability questionnaire (OQ2 = Oculus Quest 2)

Results from the acceptability questionnaire (n = 11), a 1–7 scale, indicated an overall positive report from the physiotherapists. Figure 4 shows more details about each item’s responses. All the physiotherapists found OQ2 easy to wear (Q1 – mean ± std = 6.2 ± 0.9) and easy to control (Q4 – mean ± std = 6.0 ± 0.9); moreover, all the physiotherapists felt very confident (Q2 – mean ± std = 6.1 ± 0.9) and safe in OQ2 (Q3 – mean ± std = 6.0 ± 0.9). The majority of physiotherapists (73%) enjoyed the experience in OQ2 (Q6 – mean ± std = 5.2 ± 1.7), and most (82%) agreed, somewhat agreed, or strongly agreed that they found OQ2 comfortable (Q5 – mean ± std = 5.6 ± 1.1). Although 36% of physiotherapists showed disagreement, most (64%) would like to use OQ2 weekly (Q7 – mean ± std = 4.5 ± 2.5), and they would recommend OQ2 to a colleague (Q8 – mean ± std = 4.6 ± 2.1). Only 18% of physiotherapists felt a sense of wellness after using OQ2 (Q9 – mean ± std = 3.3 ± 1.4), and most (82%) showed a positive opinion on the size of OQ2 (Q11 – mean ± std = 5.6 ± 2.2). 45% of physiotherapists showed equal agreement and disagreement on expectations on OQ2 (Q10 – mean ± std = 4.0 ± 1.8). 55% of physiotherapists thought that gaining benefits by using OQ2 regularly is possible (Q12 – mean ± std = 4.4 ± 1.8), and most (73%) agreed, somewhat agreed, or strongly agreed that OQ2 should be more accessible to those who need it (Q13 – mean ± std = 5.8 ± 1.9).

### Usability questionnaire

**Fig. 5:**
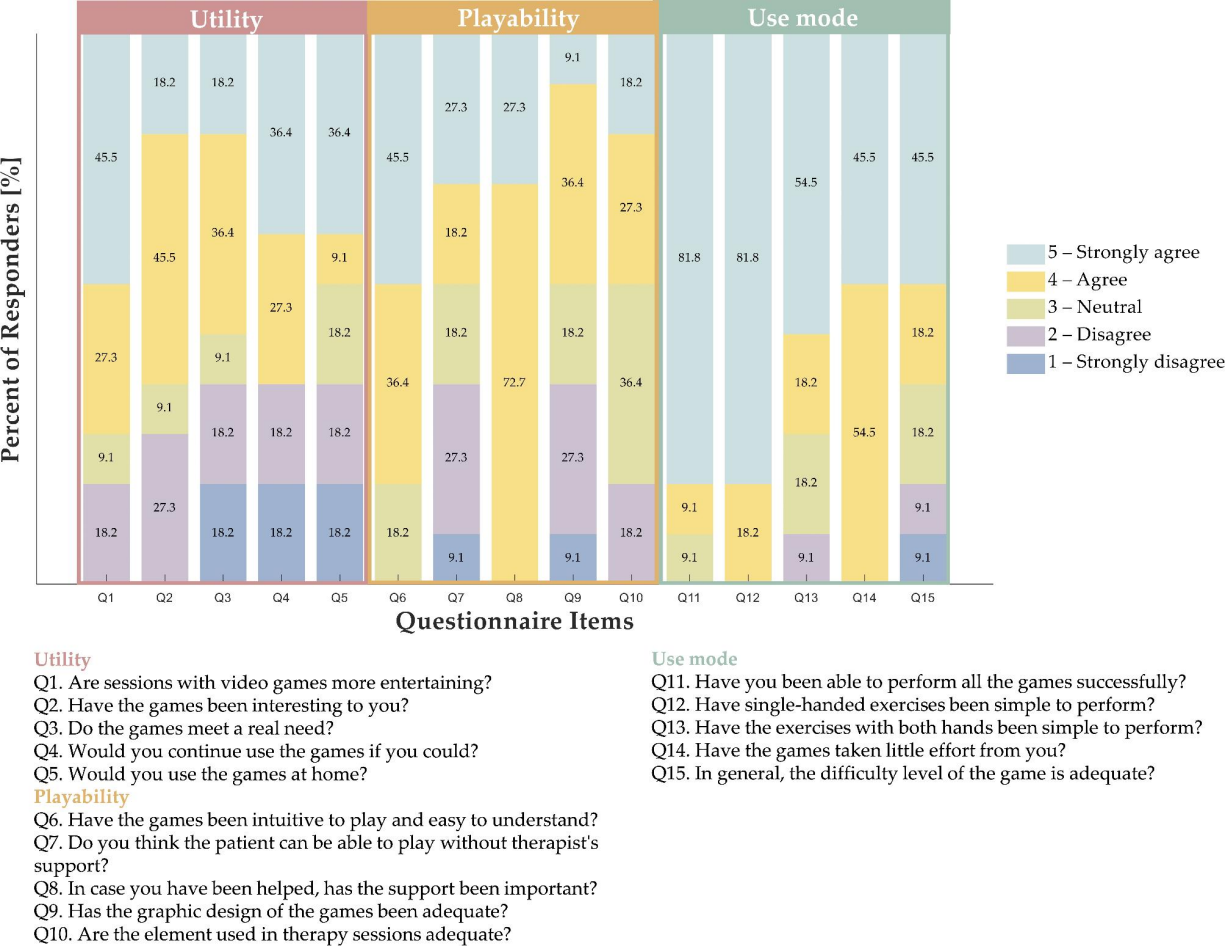
Physiotherapists’ ratings on the usability questionnaire

Results from the usability questionnaire (n = 11), a 1–5 scale, are reported in Fig. 5. Most physiotherapists (73%) agreed and strongly agreed that sessions with video games are more entertaining (Q1 – mean ± std = 4.0 ± 1.2). 63.7% of physiotherapists found the developed VR application interesting (Q2 – mean ± std = 3.5 ± 1.1) enough to want to continue using the games if they could (Q4 – mean ± std = 3.4 ± 1.6). 55% of physiotherapists agreed and strongly agreed that the physical therapy protocol developed in an VR environment meets a real need for patients with shoulder diseases (Q3 – mean ± std = 3.2 ± 1.5). Considering the end users of OQ2 and the ad hoc developed VR app, only 45% of physiotherapists would use the VR system for home rehabilitation (Q5 – mean ± std = 3.3 ± 1.6).

All the physiotherapists agreed and strongly agreed that the help was crucial for using OQ2 and playing the VR app of the physical rehabilitation program (Q8 – mean ± std = 4.3 ± 0.5). The majority of physiotherapists (82%) agreed and strongly agreed that the games were intuitive to play and easy to understand (Q6 – mean ± std = 4.3 ± 0.8). However, only 45% of physiotherapists agreed and strongly agreed that patients can be able to play without the therapist’s support (Q7 – mean ± std = 3.3 ± 1.4), the graphic design of the games was adequate (Q9 – mean ± std = 3.1 ± 1.2), and the elements used in the therapy sessions were adequate (Q10 – mean ± std = 3.4 ± 1.0).

All the physiotherapists found that the games required little effort from their (Q14 – mean ± std = 4.4 ± 0.5), and the single-handed exercises were easy to perform (Q12 – mean ± std = 4.8 ± 0.4). The exercises with both hands were simple for 73% of physiotherapists (Q13 – mean ± std = 4.2 ± 1.1). The majority of physiotherapists (82%) were able to perform the physical therapy program successfully (Q11 – mean ± std = 4.7 ± 0.6), and most of them (64%) found the difficulty level of the game adequate (Q15 – mean ± std = 3.8 ± 1.4).

### Appropriateness questionnaire

**Fig. 6:**
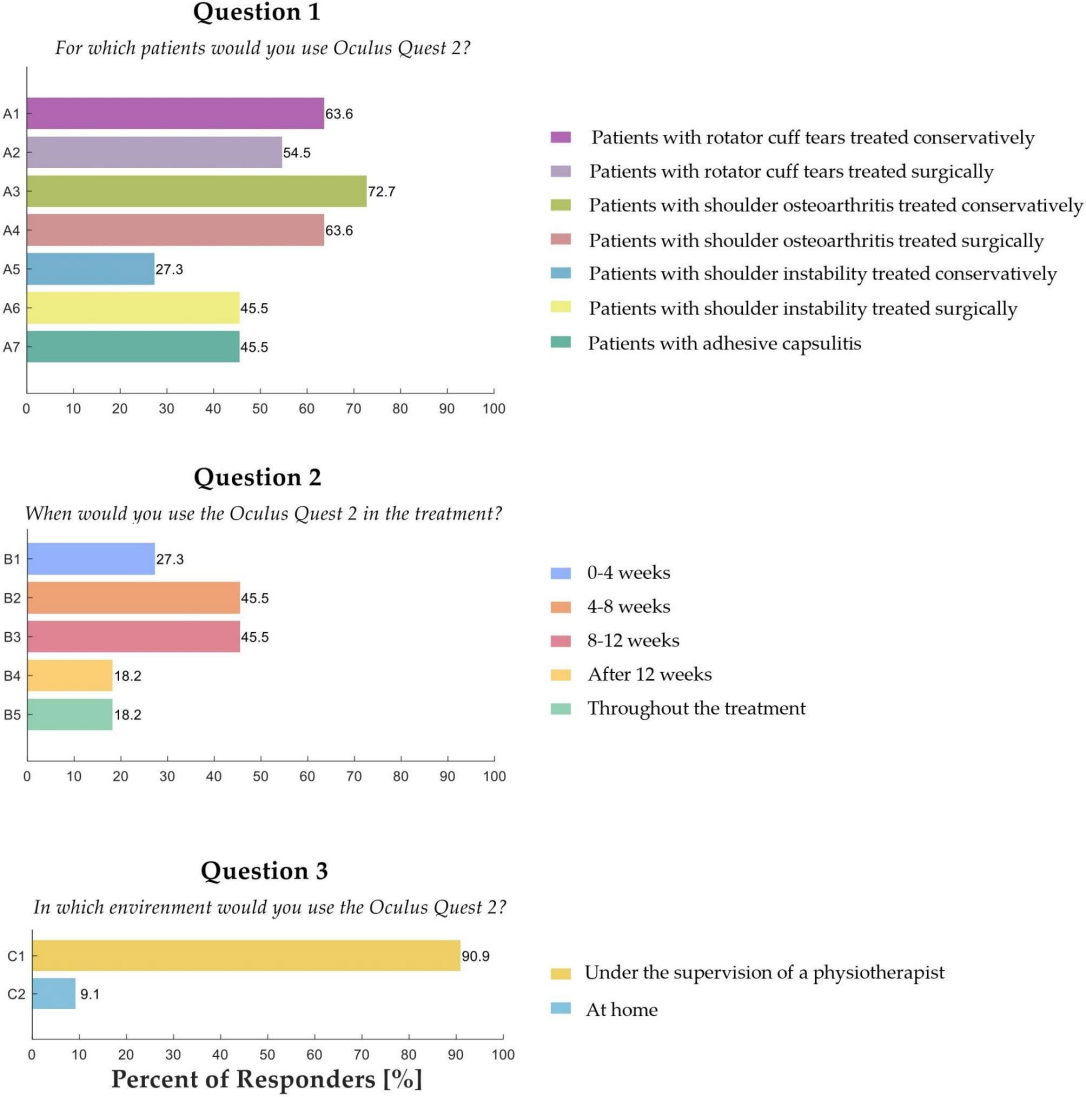
Physiotherapists’ ratings on the usability questionnaire

Figure 6 shows the appropriateness questionnaire results for the multiple-choice questions (Question 1: For which patients would you use OQ2; Question 2: When would you use the OQ2 in the treatment? Question 3: In which environment would you use the OQ2?).

Results showed that 63.6% of the physiotherapists would like to use OQ2 for patients with rotator cuff tears treated conservatively or with shoulder osteoarthritis after surgical treatment. Concerning the treatment phase, 45.5% of the physiotherapists would like to use the VR application during the mid-rehabilitation phase (4–8 weeks) and the late rehabilitation phase (8–12 weeks). Moreover, 91% of physiotherapists think it would be best for patients to use OQ2 under the supervision of a therapist and not independently in a home setting.

The open-ended question was, “*What do you think about your experience with Oculus Quest 2? Do you have any suggestions?*”. The experimenters systematically collected and analyzed the answers of the physiotherapists after outlining and explaining to them the categories to be referred to, namely exercise correctness, graphic design, autonomy in use, and engagement. 63% of physiotherapists argued about the exercises’ correctness by highlighting the need to perform head and trunk rotations to complete the exercise. Such a condition did not correctly isolate the shoulder flexion-extension, adduction-abduction, and internal-external rotations. Physiotherapists have found that performing the exercises only in a standing position could fatigue the patient. Overall, physiotherapists rated the graphics of the developed VR app as elementary but pleasant. In designing the app, the physical therapists suggested adding an avatar that mirrors the patients’ figure and is capable of guiding them in performing the exercises.

## Discussion

This study provides physiotherapists’ perspectives on the use of a VR application for executing a rehabilitation protocol in the context of shoulder musculoskeletal pathologies. In developing a VR environment designed for medical applications is essential to provide relevant insights to developers to reach high standards before using VR systems with patients. For this reason, the rationale behind the present study was to investigate several aspects such as utility, playability, use mode, ease of use, ease of understanding, safety, comfort, size, and wearability, considering the opinions of experts in physiotherapy of the shoulder with orthopaedic diseases preventively. Concerning acceptability, the study results indicated that physiotherapists have positive opinions about the application of the VR system for shoulder rehabilitation. Indeed, physiotherapists found it comfortable, safe, and easy to use. Concerning usability, physiotherapists affirmed that the VR system is interesting, intuitive, and easy to understand. However, regarding playability, not all physiotherapists think that the final VR application fulfills the requirements of the end users. The main difficulty encountered was the need to perform accessory movements (i.e., head and trunk rotation, cervical extension), thus not allowing proper isolation of shoulder movements and potentially affecting the rehabilitation treatment. Moreover, many physiotherapists believe that patients cannot use it independently without external support to prevent the execution of incorrect movements unless feedback is added for patients on the accuracy of exercise execution. From this observation emerged the need to provide a more precise description of the execution of the protocol and, most importantly, to provide the therapist with feedback so the patient can be corrected. Data provided by the VR system could be used to evaluate shoulder movements during exercises and define performance indices evaluating the execution level of the protocol. Moreover, adding an avatar of the patient could have positive effects on engagement, autonomy in use, and correctness in performing the exercise. In fact, some physiotherapists believe that home use may be compromised by the risk of incorrectly performing physiotherapy exercises. Concerning appropriateness, physiotherapists found that OQ2 and the VR rehabilitation program are appropriate for shoulder pathologies, especially for patients with shoulder osteoarthritis treated conservatively. Indeed, the VR system needs active or assisted active movements, and a post-surgical patient can first perform only passive movements while active movements are generally delayed. Also, other studies evaluated the usability of VR systems for rehabilitative purposes, although in clinical applications other than the shoulder, such as post-surgery elbow rehabilitation, stroke, and Parkinson’s diseases [38–40]. Similar to our study, the usability metrics investigated concerned the intuitiveness and clarity of the game, ease of use, comfort, enjoyment, utility, and use mode. All these investigations served ad support to provide essential insights into the future developments of the proposed VR application.

In recent years, few studies have investigated the use of VR systems to improve joint functionality in patients with shoulder musculoskeletal diseases [12, 20, 33, 40, 41]. Although these studies are not fully comparable because of the heterogeneity of the type of VR systems used and the custom-made VR application, most researchers have reported the beneficial effects of VR on the rehabilitation of shoulder musculoskeletal pathologies [12, 20, 33, 40, 41]. However, several studies have demonstrated the efficacy of VR systems in rehabilitating patients with spinal cord injuries, chronic stroke, or neurological disorders [37, 42–44]. These studies have reported the usefulness of VR in increasing the efficiency of physical therapy and enhancing motor learning after trauma.

VR systems are especially interesting and promising technology within the realm of shoulder musculoskeletal rehabilitation, yet it is not mature enough to be administered routinely to all patients. More specifically, VR offers a highly engaging experience within a computer-generated world, allowing patients to interact with virtual objects or perform certain tasks in a virtual environment. However, the current implementation of VR in the healthcare sector has several challenges that need to be addressed in order to make it suitable for widespread use in clinical practice. These challenges may include the high appropriateness of games to the type of shoulder diseases and therapeutic timelines, adherence to treatment with flexible, simple, and engaging solutions for large patient populations, and reduced user fatigue with maximization of therapeutic efficacy.

This study provided a concrete and practical perspective for using VR in rehabilitating patients with shoulder musculoskeletal pathologies. The approach followed the standards proposed in ISO 9241 − 210:2019 (Ergonomics of human-system interaction — Part 210: Human-centered design for interactive systems) and the different steps to be followed, namely the identification of end-users and context of use, the definition of user needs and system requirements, design and development of the solution, evaluation of the developed solution [45]. The study’s main contribution was to provide useful design insights for future developments based on the ideas of experienced physiotherapists in shoulder rehabilitation who are capable of understanding and delineating the difficulties patients may encounter during physical therapy rehabilitation and the benefits that they could derive from the VR application. The insights mainly concern optimizing the VR game to avoid unwanted movements other than those of the shoulder, thus facilitating autonomous use and providing feedback to users to increase the involvement and correctness of the rehabilitation exercises performed.

## Conclusions

According to the opinion of physiotherapists, OQ2 can be a useful VR system for shoulder musculoskeletal rehabilitation as it could be comfortable and engaging for patients. However, some improvements are needed to increase the possibility of using the system independently by patients. Additional studies should assess the clinical usefulness of the system on patients with shoulder musculoskeletal disorders. The use of VR in orthopaedic rehabilitation is encouraging, and evidence for its efficacy is mostly supportive. Future studies should strive to build on this impetus and further work to ensure the effectiveness of these techniques in reaching therapeutic goal settings.

## Data Availability

The data presented in this study are available on request from the corresponding author.
